# Color pan traps often catch less when there are more flowers around

**DOI:** 10.1002/ece3.7252

**Published:** 2021-03-15

**Authors:** Lars Westerberg, Hilda‐Linn Berglund, Dennis Jonason, Per Milberg

**Affiliations:** ^1^ IFM Biology, Conservation Ecology Group Linköping University Linköping Sweden

**Keywords:** Apoidea, Cetoniidae, flower colour, Lepturinae, pan trap, Syrphidae, Vespoidea

## Abstract

When assessing changes in populations of species, it is essential that the methods used to collect data have some level of precision and preferably also good accuracy. One commonly used method to collect pollinators is colour pan traps, but this method has been suggested to be biased by the abundance of surrounding flowers. The present study evaluated the relationship between pan trap catches and the frequency of flowers on small (25 m^2^) and large (2–6 ha) spatial scales. If pan traps work well, one should assume a positive relationship, that is, more insects caught when they have more food. However, in contrast, we found that catches in pan traps were often negatively affected by flower frequency. Among the six taxa evaluated, the negative bias was largest in Vespoidea and Lepturinae, while there was no bias in solitary Apoidea (Cetoniidae, Syrphidae and social Apoidea were intermediate). Furthermore, red flowers seemed to contribute most to the negative bias. There was also a tendency that the negative bias differed within the flight season and that it was higher when considering the large spatial scale compared to the small one. To conclude, pan trap catches may suffer from a negative bias due to surrounding flower frequency and color. The occurrence and magnitude of the negative bias were context and taxon dependent, and therefore difficult to adjust for. Thus, pan traps seem less suited to evaluate differences between sites and the effect of restoration, when gradients in flower density are large. Instead, it seems better suited to monitor population changes within sites, and when gradients are small.

## Graphical abstract

Colour pan traps are often recommended for pollinator monitoring. We report that pan trap catches may suffer from a negative bias due to surrounding flower frequency and color. The occurrence and magnitude of the negative bias were context and taxon dependent, and therefore difficult to adjust for. Thus, pan traps seem less suited when gradients in flower density are large. Instead, it seems better suited to monitor population changes within sites, and when gradients are small.

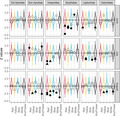

## 1 INTRODUCTION

Pollinating insects are important for many plant species and provide an essential ecosystem service to man (Aizen et al., 2009; Gallai et al., 2008). Therefore, the decline of many pollinator populations (Fitzpatrick et al., 2007; Kluster & Peduzzi, 2007) is of particular concern. The declines are often ascribed to changes in agricultural land‐use and practices during the last 50–100 years (Potts et al., 2010; Shrubb, 2003) including use of pesticides (Rundlöf et al., 2015).

To assess populations of pollinators and to monitor changes in response to habitat deterioration, as well as to conservation efforts, it is essential that the methods used to collect data on pollinator fauna have some degree of precision (i.e., that repeated sampling gives similar results). Accuracy in data is also important (i.e., to what extent it represents the populations sampled), but possibly an unrealistic goal when sampling an insect assemblage (Cooper et al., 2012; Sutherland & Parrella, 2011; Walther & Moore, 2005). The proportion of living specimens sampled will vary tremendously among species due to differing activity pattern, body size, micro‐habitat etc. So, a sampling scheme is unlikely to collect more than a single, targeted species with high accuracy. Consequently, in many cases it seems feasible to accept a consistent bias as long as relevant trends can be inferred.

Among the different methods used to survey pollinator populations, Malaise traps and suction sampling catch large numbers of insects, of which only a small proportion are pollinators (Campbell & Hanula, 2007). Transect walks and hand‐netting work well for larger flying insects like *Bombus*, while smaller and/or more mobile species are difficult to record. These are some reasons why pan traps, that specifically target pollinators by attracting them to liquid‐filled colorful pans, are considered a promising method for this important insect group (Berglund & Milberg, 2019; Falk, 2015; LeBuhn et al., 2016).

Theoretically, we expect the abundance of nectar‐feeding insects to increase with abundance of their main food resource, and a number of observational studies have confirmed this (e.g., Ebeling et al., 2008; Franzén & Nilsson, 2008; Hegland & Boeke, 2006; Pengelly & Cartar, 2010; Potts et al., 2003; Sajjad et al., 2010; Sjödin, 2006). Because of that relationship, we might assume that catches in pan traps should be linearly positive to density of flowers. In contrast, if there is competition between flowers and the pan traps, that is, a negative bias in catches, the relationship would not be linearly positive. There could either be a lack of relationship between flower density and pollinator numbers, or a negative one (or a non‐linear one). Also, when there is a high density of flowers, the probability of a flower visit might decrease: a phenomenon termed pollinator limitation (Pettersson & Sjödin, 2000; Sih & Baltus, 1987; Steven et al., 2003). This phenomenon is likely also to affect the probability of a color trap visit. Finally, the chance of a catch might increase when flowers are few or non‐existent because pollinators may concentrate on flowers in resource‐poor environments (Kleijn et al., 2011).

Some studies have reported smaller pan trap catches when there are more flowers around (Baum & Wallen, 2011; Cane et al., 2000; Kovács‐Hostyánszki et al., 2011; Mayer, 2005; Roulston et al., 2007; Templ et al., 2019; Wilson et al., 2008), others have recorded the opposite (Wood et al., 2015). There might also be a bias due to flower colors, as insect species might have color preferences (Campbell et al., 2010; Reverté et al., 2016), a feature seen also in multi‐colored pan‐trapping where different pan trap colors are preferred by different taxa (Campbell & Hanula, 2007; Cane et al., 2000; Toler et al., 2005; Wilson et al., 2008). Blue, white, and yellow are frequently used colors, most often in combination (Campbell & Hanula, 2007; Saunders & Luck, 2013; Toler et al., 2005; Wilson et al., 2008). In terms of bias through competition and pollinator limitation, it seems plausible that the colors of the flowers around the pan traps may also affect the catches in the pan traps, for example, if an insect taxon prefers blue flower over blue pan traps. However, Toler et al. (2005) found no effect of the colors of surrounding flowers on the catches. Given the somewhat conflicting reports cited above, it remains unclear if and when a flower abundance bias exists.

The aim of the present study was to relate pan trap catches of pollinating insects (solitary Apoidea, social Apoidea, Vespoidea, Lepturinae, Cetoniidae, Syrphidae) to the flower frequency, both in the nearest surroundings of the pan traps (25 m^2^) and on a larger spatial scale (2–6 ha). The study system was clear‐cuts in production forest, selected to represent a gradient in flower frequency, that were sampled three times during the flight season.

## 2 MATERIAL & METHODS

### 2.1 Study sites

The study was conducted in 2015 the province of Östergötland, southern Sweden. The landscape in the study area consists mainly of coniferous forest, but there are also bogs, lakes, small patches of seminatural grasslands, and arable fields (Ibbe et al., 2011; Milberg et al., 2019).

Twelve clear‐cuts were selected for the study, each had an area of 2–6 ha, had been logged 4–6 years prior to the study, and were situated at a minimum distance of 300 m from nearest seminatural grasslands. Six of them had been marked as coniferous forests on maps from the 1870s when the other six were marked as meadows. Clear‐cuts on former meadows have higher amounts of herbs than clear‐cuts which were formerly forests (Jonason et al., 2014, 2016) and have more butterflies and burnet moths (Bergman et al., 2020; Blixt et al., 2015). Hence, our site selection strategy covered the wide range of flower abundances that occur on clear‐cuts in the study area, and we assume this range would allow the study of effects of flower abundance on pollinator catches.

### 2.2 Pan traps

The pans used to collect pollinators were painted in one of the following colors: blue, white and yellow with UV‐reflecting‐color (Soppec, Sylva mark fluo marker). The pans had a diameter of 8.7 cm, a volume of 0.5 L and were filled with non‐toxic propylene glycol (40% concentration), to conserve the pollinators and to decrease the surface tension. A small opening (4 mm in diameter) at the top of each bowl was made to ensure that rainwater could drain. One set of pan trap consisted of three pans, one in each color, placed on a steel stick.

Four sets of pan traps were placed in each clear‐cut, in the same height as the vegetation and in places that were considered representative for the clear‐cut. As clear‐cuts differed in size, so did the distance between the four sets of traps. During the main flight period of most pollinating insects, three sampling periods were conducted: in the beginnings of June, beginning of July, and beginning of August. Each period lasted for 1 week and had at least some days with more than 17°C and wind velocity less than four on the Beaufort scale (cf. Wikström et al., 2009). The pans were covered with caps between collecting periods. When a new collecting period started, some sets of pan traps were moved—at most 30 cm—or some of the vegetation was removed to prevent overgrowth.

In total, there were 48 set of pan traps collecting during each period, but a few sets of pan traps had been knocked down by animals and were therefore excluded (1, 1, and 2 during the first, second and third period, respectively). In addition, a single blue pan went missing during the second period. For this reason, the catch was expressed as number of specimens per trap.

Four taxonomic groups dominated the catches—Aculeata, Lepturinae, Cetoniidae and Syrphidae—and they are the focus of the current study. Aculeata was subdivided as solitary Apoidea, social Apoidea (*Bombus* spp. *Apis mellifera*), and Vespoidea (including one species of Chrysidoidea). Other insects caught, that are not considered here, were mainly small Coleoptera, small Lepidoptera, and Symphyta.

### 2.3 Flower frequency

**FIGURE 1:**
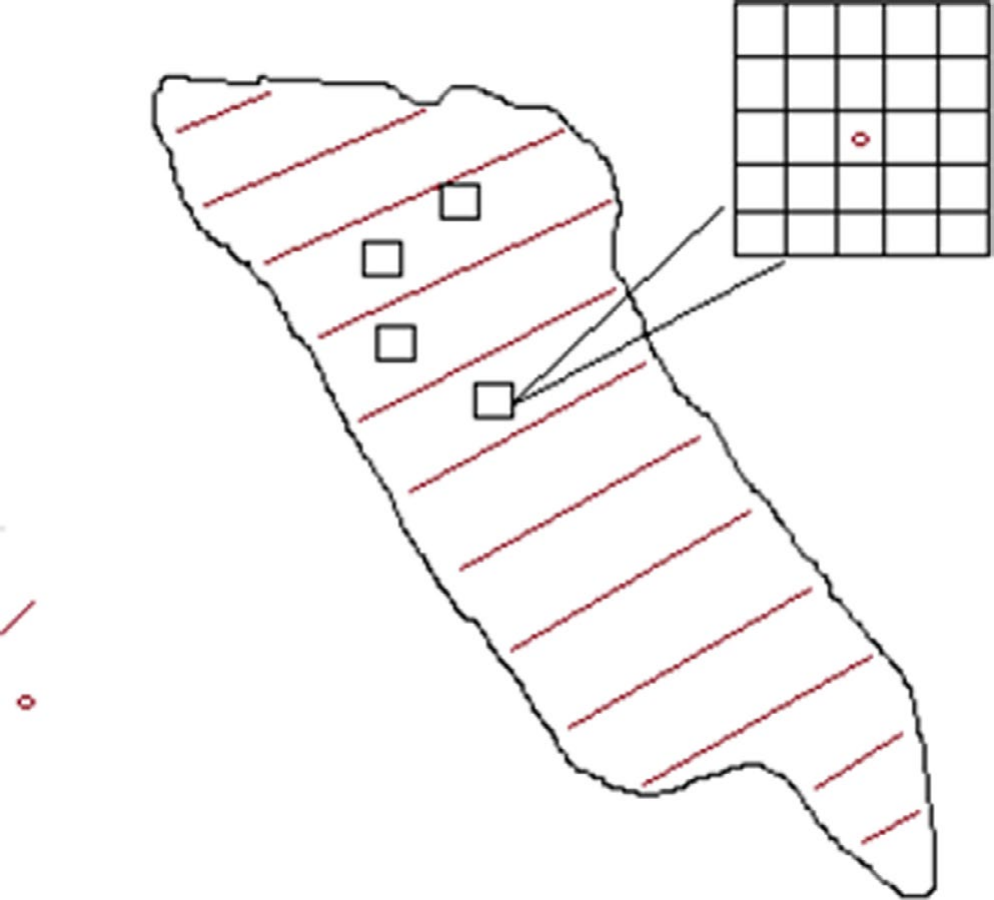
An example of a clear‐cut with pan traps and transects indicated. Over the whole clear‐cut, there were c. 100 photographs taken along the transects, with an additional 25 photographs around each of the four sets of pan traps. All pictures were photographed over a 1‐m square lying on the ground

The clear‐cuts were photographed in conjunction with each collecting period to estimate flower frequency. A 1 m^2^ square was placed on the ground and photographed from above. Around each pan trap, at least 25 such 1 m^2^ squares were photographed reflecting small‐scale flower abundance (“trap scale”). An additional at least 100 pictures were systematically distributed along transects over the whole clear‐cut reflecting large‐scale (“clear‐cut scale”) flower abundance (Figure 1). As the size of clear‐cuts varied, the distance between photographs taken varied for the clear‐cut scale. Photographs were taken during each collecting period, or at most 5 days before or after. The camera used was a Canon EOS 550D with 18–55 mm lens; we used 18 mm and took photographs with a flash light at a height of ca. 160 cm.

All 8,048 photographs taken were inspected to see if they held flowers within the 1‐m^2^ square and if so of which colors (red, yellow, blue, and white). The frequencies of colors (a) around set of pan traps, and (b) on clear‐cuts (Figure 1) were expressed as the odds for the color occurring in a square meter plot: (0.5 + *p*)/(0.5 + (1 − *p*)), where *p* = frequency of photos with the color. Also, we calculated the odds for flower of any color occurring in a plot.

### 2.4 Data analyses

Fitting a complex model to the complete data set for hypothesis testing is challenging. Generalized linear mixed models are limited by the low sample size, especially at high model complexity with few replicates for each combination. Penalizing methods such as lasso, ridge, or elastic net regression models could be an alternative as they shrink variables and interactions with less support toward zero. However, models with low sample size are heavily penalized and n‐way interactions are difficult to fit. Therefore, we chose to conduct several simple GLM, and to compare these by using a test statistic as “effect size.”

We conducted regression analyses (GLM with poisson distribution and log link) using the glm()‐function in R (R Core Team, 2020): with odds of flowers occurring in a 1‐m^2^ explaining number of individuals caught per trap‐group. As the data often did not comply with a Poisson distribution, we used quasi‐poisson distribution that fits an additional dispersion parameter. Models were usually moderately under or over‐dispersed. As the data on odds were skewed, they were ln‐transformed before analyses. We evaluated red, yellow, blue and white flowers separately, as well as the odds of any flower occurring in 1 m^2^. In total, we conducted 180 models (six taxonomic groups, five colors; three sampling periods; two spatial scales).

These 180 models were compared by their *Z*‐value (the ratio of the regression coefficient and standard error, adjusted by the dispersion parameter). As sample size is constant within scales, we used *Z*‐value as a proxy for effect size, facilitating comparison between flower color, taxa and month. Models with high *Z*‐value or low negative Z‐value, depending on the direction of the relationship, indicate a stronger association between flowers and trap catch. Using *Z*‐values as an estimate of effect size enables the direct comparison of, for example, different taxa and colors (which would not be possible using regression coefficients). More specifically, we used the distribution of *Z*‐values as a key feature for two reasons. First, as there were only 12 clear‐cuts, the statistical power of individual models was low making an overall analysis more relevant than individual analyses (i.e., reduce the risk for type II error). Second, the shape of the distribution of *Z*‐values can be related to the expected outcomes in case of unbiased and biased catches. We can envision three outcomes:

- Outcome 1: A positive relationship between catches and flower frequency, that is, the data reflect the assumed increased insect abundance with increased flower abundance.
- Outcome 2: No relationship between catches and flower frequency; either the assumed relationship is faulty or there is a bias in catches that prevent the assumed relationship being manifested in data.
- Outcome 3: A negative relationship between catches and flower frequency; the decreasing abundance with flower frequency suggests a negative bias in pan trap catches.

*Z*‐values were explored in two different ways, all involving a randomization procedure to produce a comparison of “no effect” (i.e., outcome 2 above). In the latter, 499 permutations of color frequency were conducted. Permutations were restricted to within (a) insect taxon, (b) flower color and (c) collecting period (i.e., 499 permutations for each of the 90 combinations). These three, together with spatial scale, were our focus in the following comparisons. First, the *Z*‐values were compared with their corresponding *Z*‐values based on permuted data. The observed *Z*‐value was considered “significantly positive” if it was larger than 95% of the permuted *Z*‐values, and “significantly negative” if smaller than 5% of the permutes *Z*‐values. Second, the cumulative distribution of observed *Z*‐values was visually compared with those based on permuted data by grouping together *Z*‐values per (a) taxa (*N* = 30 *Z*‐values per taxon), (b) flower colors (*N* = 30), (c) spatial scales (*N* = 75), and (d) time periods (*N* = 50). All procedures (permutations and postprocessing) were developed in and carried out using R and base R functions (R Core Team, 2020).

## 3 RESULTS

The total number of insects encountered in the six taxonomic groups was 2,192. Solitary Apoidea was the most species‐rich group (69 species), followed by Syrphidae (49), Vespoidea (21), Social Apoidea (12), Lepturinae (7), and Cetoniidae (2 taxa). Social and solitary Apoidea were relatively evenly distributed among the three sampling periods, while Lepturinae and Vespoidea increased and Syrphidae & Cetoniidae decreased over the season (data not shown).

**FIGURE 2:**
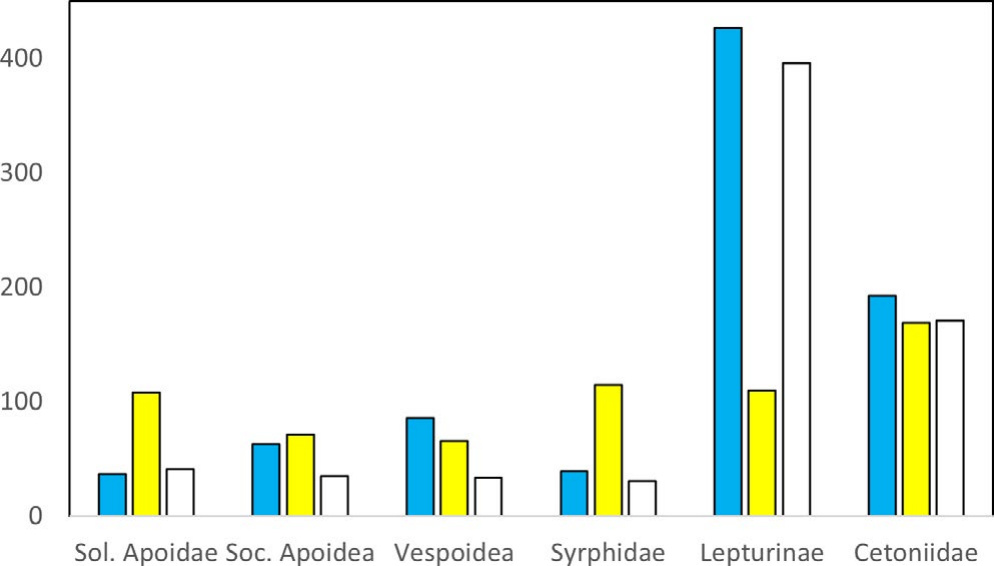
Number of specimens caught in pan traps of different colors. Data merged over four traps, on each of twelve clear‐cuts, and three collecting periods (early June, early July, early August)

Individuals of social Apoidea, Vespoidea, and Cetoniidae caught in the pan traps were relatively evenly distributed among pan trap colors. Solitary Apoidea and Syrphidae were mainly caught in yellow traps whereas Lepturinae avoided yellow (Figure 2).

**FIGURE 3:**
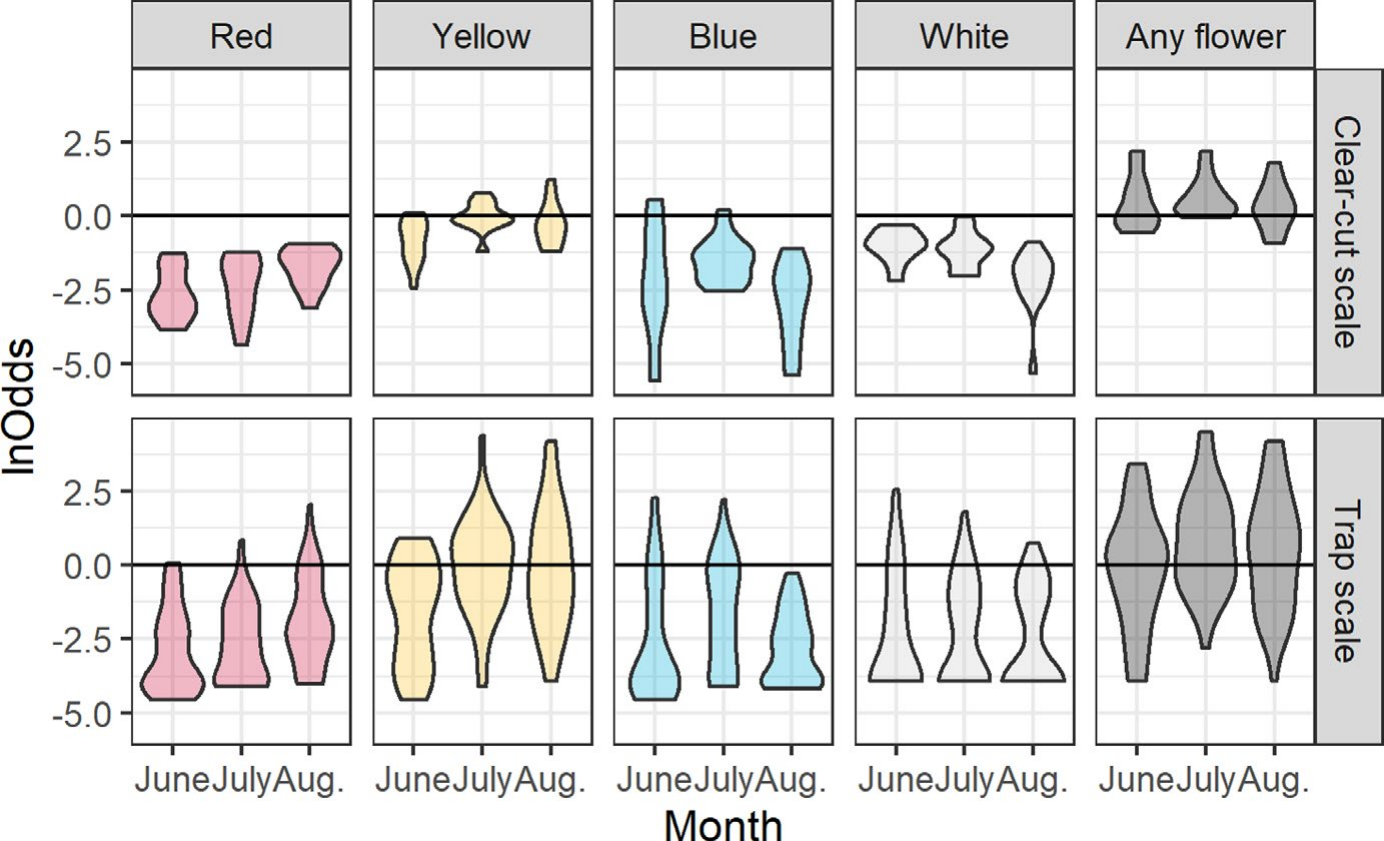
Violin plots (mirrored density plots) of ln(odds of flower per m^−2^) at the clear‐cut scale (2–6 ha) and the trap scale (25 m^2^) and per collecting period (early June, early July, early August)

The odds of finding a flower per m^−2^ were slightly higher in early July, but there were deviations when considering individual colors (Figure 3).

**FIGURE 4:**
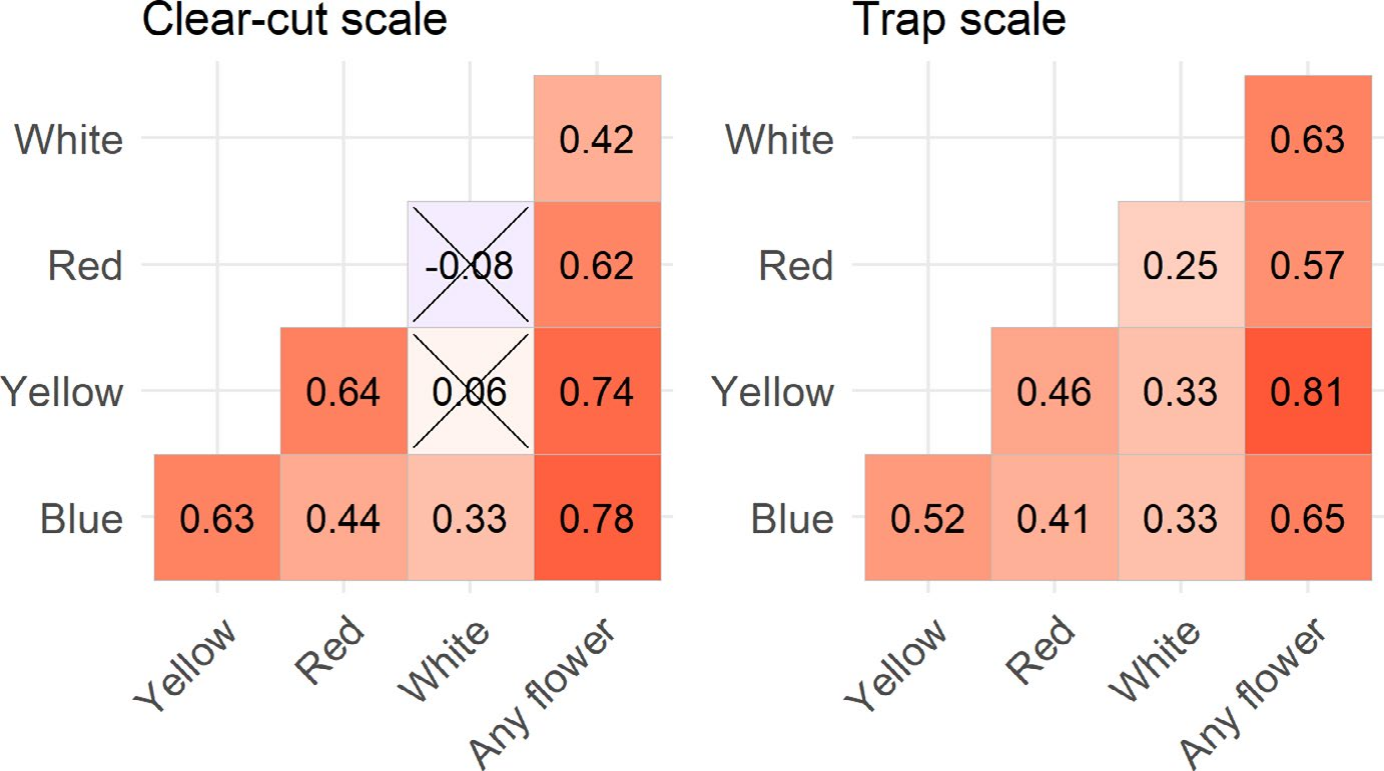
Pearson's correlation of ln(odds of flower in m^2^) in data from the trap scale (25 m^2^ around pan‐traps) and on the clear‐cut scale (2–6 ha). There were 12 clear‐cuts and three collecting periods. On each clear‐cut, there were four trap locations. All correlations were significant (*p* < 0.05) except the two crossed out

The correlations between the ln(odds for recording a flower per m^2^) were generally high among colors (Figure 4).

### 3.1 *Z*‐values

**FIGURE 5:**
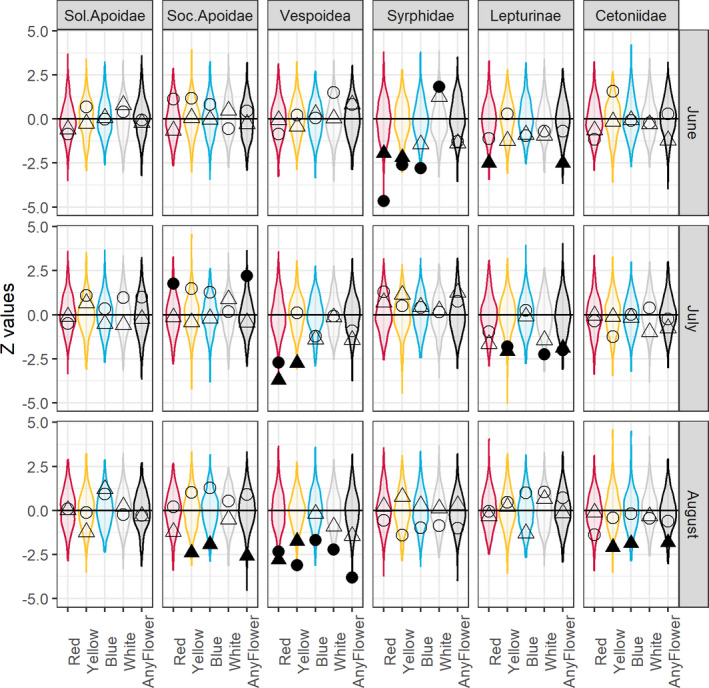
*Z*‐values from GLM analyses relating abundance of insects to flower occurrence. Symbols represent *Z*‐values from observed data while the violin plot (a mirrored density plot) displays the distribution of *Z*‐values of the permuted data. Circles are *Z*‐value for trap scale data and triangles are clear‐cut scale data. Filled symbols indicate that the observed association is more extreme (higher than 95%, or lower than 5%) than the permuted *Z*‐values

In the majority of observed associations between numbers caught and frequency of flowers, the *Z*‐value was below zero (73% for clear‐cut scale and 56% for trap scale). Of the 180 *Z*‐values, many were well within the probability distribution of *Z*‐values based on permuted data (Figure 5). However, 32 of them (18%) were unlikely to have emerged from permuted data (*p* < 0.05), and of these three were positive (2%) while 29 were negative (16%). The three cases with positive relationship between animals caught and flower frequency only involved the small spatial scale: social Apoidea (any flower, July), Syrphidae (white, June) and Cetoniidae (yellow, June). The 29 negative relationships involved all taxonomic groups except solitary Apoidea (3 social Apoidea, 11 Vespoidea, 5 Syrphidae, 7 Lepturinae, 3 Cetoniidae), and occurred at both trap (12) and clear‐cut scales (17), and with all colors (8 red, 9 yellow, 3 blue, 2 white, 7 any flower). Hence, relationships between catches and flower frequency were mainly insignificant or negative (Figure 5).

### 3.2 Cumulative distribution of *Z*‐values

**FIGURE 6:**
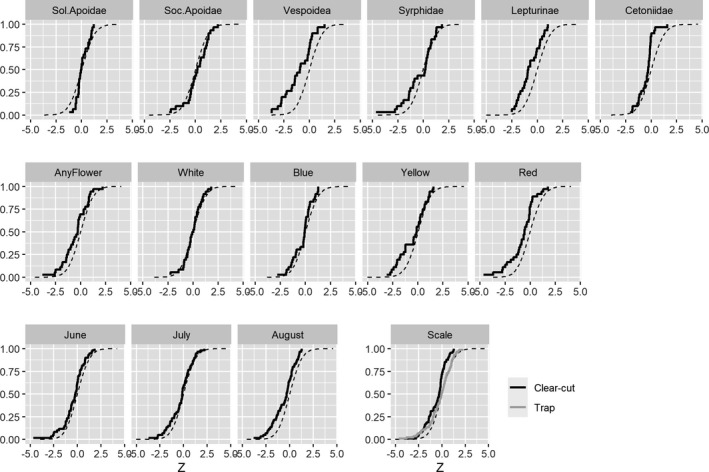
Cumulative distribution of *Z*‐values from GLM analyses relating insect abundance to flower frequency. Solid line is based on observed data while dotted line shows *Z*‐values based on permuted flower data

Compiling groups of *Z*‐values against the backdrop of permutation outcomes (Figure 6) revealed the following patterns.

#### 3.2.1 Taxonomic groups

Vespoidea deviated most from the expected distribution (Figure 6). Lepturinea also showed generally negative relationships, and Syrphidae did so too but only an the lower tail of the curve (Figure 6). For the solitary and social Apoidea, the curves followed the permuted one, and for Cetoniidae, only the upper parts of the curve were biased (i.e., much fewer positive *Z*‐values than expected; Figure 6).

#### 3.2.2 Colors

Red was the color most affected by negative bias while white and blue seemed unaffected (Figure 6). Any flower was consistently negatively biased while yellow was biased only for negative *Z*‐values (Figure 6).

#### 3.2.3 Collecting periods

The three collecting periods all displayed an overall negative bias while with August slightly more than the two other occasions (Figure 6).

#### 3.2.4 Spatial scales

Both spatial scales, and especially the large clear‐cut scale, were negatively biased (Figure 6).

## 4 DISCUSSION

When using pan traps, one expects the catch to reflect the size of targeted insect populations. It is difficult, however, to assemble an independent, accurate sample of an insect population to challenge this basic premise. That is why we chose to address the accuracy of pan traps by relating catches to food availability, or more precisely flower frequency per m^2^. Our study therefore rests on the assumption that more flowers often means higher densities of nectar‐searching insects. Although there are many studies observing more insects when flowers are more abundant (Ebeling et al., 2008; Franzén & Nilsson, 2008; Hegland & Boeke, 2006; Pengelly & Cartar, 2010; Potts et al., 2003; Sajjad et al., 2010; Sjödin, 2006), including in the current study system,^1^ there can be exceptions. Some species might be more affected by the availability of host plants for oviposition (e.g., butterflies in the current study system, Ibbe et al., 2011) or the occurrence of breeding sites (Heneberg et al., 2016; Westerfelt et al., 2018), or might not be able to, or not sufficiently mobile to, exploit the full variety of flowers in the vicinity (Greenleaf et al., 2007; Osborne et al., 2008; Zurbuchen et al., 2010). Hence, it is entirely possible that there might be cases with no relationship between food and abundance, or even a negative one, and that is important to remember when evaluating the results. Ignoring this caveat for a while, we expected pan trap catches to increase with flower frequency, which would be manifested by (a) more positive than negative *Z*‐values, (b) more significant positive than significant negative *Z*‐values, and (c) positive biases in the distribution of *Z*‐values. None of this could be seen in our data (Figures 5, 6). Furthermore, there was not much support for the null hypothesis (i.e., no effect on flower frequency on the trap catch) with only a few cases (white flowers; solitary and social Apoidea) where *Z*‐values from observed and permuted data coincided. The third outcome—that catches decreased with increasing flowers—found support in many cases. First, there were more negative *Z*‐values than positive; second, there were more significant negative *Z*‐values than corresponding positive ones; and third, the distribution of *Z*‐values was often fully or partly to the left of the curve based on permuted data. We believe this is mainly due to a bias, rather than to a high prevalence of insect abundance decreasing with flower abundance.

Given the negative bias in pan trap catches, this method to sample pollinators seems less suited to evaluate differences between sites and the effect of restoration, when gradients in flower density are large. In the current study, the frequency of flowers around traps in square meter plots ranged from 9% to 100% (see supplementary data) that is, a substantial gradient in flower frequency. Possibly, pan traps are better suited to monitor population changes within sites, and when gradients are small.

The actual mechanisms involved that create the negative bias remains unclear. Is there competition between flowers and the pan traps, as previous studies have suggested (Cane et al., 2000; Roulston et al., 2007; Wilson et al., 2008), that is, that pollinators prefer flowers over traps? Or is it a consequence of pollinator limitation at higher flower densities (Delmas et al., 2015; Pettersson & Sjödin, 2000; Sih & Baltus, 1987; Steven et al., 2003), that is, that visitation rates to both trap and flower decrease? Or is it a consequence of traps “over‐performing” at low flower densities? For example, pollinators aggregate at the few resources available in a resource‐poor landscape (see Kleijn et al., 2011) and visitation rates to rare flowers might be high (Wagenius & Lyon, 2010). These two observations suggest that traps might be more often visited in situations when flowers are scant or non‐existent. Fundamentally, the mechanism involves either the relationship between insect populations and their resources or the relationship between trap catches and insect populations, or most likely a combination of both. As pan traps often function in the same way as flowers, separating them would require a more elaborate study design than ours.

If a bias is consistent, it can be adjusted for. However, there seems to be little justification for such adjustments given that negative bias depended on flower colors (red being worst and white being unbiased), on when sampling occurred, and on spatial scale considered. Furthermore, a positive bias in resource‐poor habitats and a negative bias in resource‐rich habitats would mean there is a risk that pan traps will overestimate populations when small and declining and at the same time underestimate when populations are large or increasing. Hence, bias varies along the resource gradient, further complicating any adjustment and also generally increasing the risk of type II error (non‐rejection of false null hypothesis) when assessing a population trend or assessing an intervention.

### 4.1 Taxa

The only taxon that seemed not to be negatively biased was solitary Apoidae, and the reason might be that the populations of species occurring on clear‐cuts are often governed by nest site availability (Heneberg et al., 2016; Westerfelt et al., 2018). Furthermore, the mobility of solitary Apoidea tends to be modest, which limits the area an individual's foraging (Greenleaf et al., 2007; Osborne et al., 2008; Wright et al., 2015; Zurbuchen et al., 2010). Thus, it seems reasonable that these factors, rather than flower frequency, determine trap catch among solitary Apoidea.

Social Apoidea, that is, bumblebees and honeybee, have several unique features among the studied taxa. With colonies and division of labor, they can be quite numerous among flower‐visiting species. They tend to fly longer distances in search of food than most insects (Osborne et al., 2008). Their ability to communicate (von Frisch & Chadwick, 1967) should also lead them to aggregate in flower‐rich areas. *Apis* and *Bombus* are so called flower constants, that is, they forage on a unique floral species as long as it offers a profitable reward before switching to another (Chittka et al., 1999). These attributes of social Apoidea suggest the potential to aggregate in flower‐rich spots rather than in flower‐rich sites. Our data support this assumption as 12 of 15 cases had positive *Z*‐values on the small trap scale (25 m^2^), but only 2 of 15 on the large clear‐cut scale (2–4 ha).

Vespoidea had the strongest negative bias of the six taxa considered, and a consistently negative one. In fact, almost all its *Z*‐values were negative. Why this is so, remains unclear but it is worth noting many species are predators. In a study comparing two trapping methods, Rubene et al. (2015) concluded that pan trap worked best for pollen‐collecting bees while window traps were preferred for wasps.

Lepturinae and Vespoidea showed consistent negative biases while other groups were only partly biased: Syrphidae was negatively biased only at the lower end of the scale while Cetoniidae only at the upper end of the scale. This suggests that the bias is context dependent rather than consistent.

### 4.2 Colors of flowers

We assumed that color of flowers around traps might affect catches as species, as well as higher taxa, have been reported to have flower color preferences (Abrahamczyk et al., 2010; Campbell & Hanula, 2007; Joshi et al., 2015; Moreira et al., 2016; Toler et al., 2005; Wilhelmsson, 2014; Wilson et al., 2008). We confirmed this assumption of differing bias due to colors: red flowers lead to a consistent negative bias while white flowers, and to some extent blue, generated *Z*‐values in line with those based on permuted data (i.e., supporting the null hypothesis of no relationship between flower frequency and insects caught). Yellow flowers partially added to negative bias at the lower end of the scale.

When looking at cases with a known preference of a color, the results are conflicting and difficult to interpret. Syrphidae prefers yellow flowers (Klecka et al., 2018; Wilhelmsson, 2014) and more individuals were found in yellow pans (Figure 2) but had generally a non‐response or negative response to yellow flowers and flowers in general when analyzing subsets of data. Solitary Apoidea also preferred yellow (Figure 2) but catches were unaffected by yellow flowers during individual months (Figure 5). Finally, Lepturinae avoids yellow (Figure 2, Wilhelmsson, 2014) and was unaffected or negatively affected by yellow flowers (Figure 5). To conclude, although we documented an effect on pan trap catches of the color of surrounding flowers, it was not apparently related to the color preferences of the insect groups, perhaps because of too few individuals in subsets of data.

There are two obvious challenges when interpreting flower color effects. First, flower color preferences can be species‐, genus‐, or family‐specific (e.g., Hall, 2016; Klecka et al., 2018), so merging numerous responses of several taxa is likely to hide preferences. Second, we classified the flower to color according to human vision, which in many ways differ from how insects perceive colors (Chittka & Raine, 2006). The three insect orders considered here (Hymenoptera, Diptera, Coleoptera) all have several photoreceptors enabling them to distinguish different colors (Briscoe & Chittka, 2001). Generally, insects are sensitive to light with short wavelengths (ultraviolet, not visible to humans), but often lack receptors sensitive to long wavelengths (red). This is probably why red pan traps attract few insects and are rarely used (Campbell & Hanula, 2007). On the other hand, in contrast to pan traps, flowers rarely reflect monochromatic light but rather a mixture. So red flowers might not be detected by insects by reflecting red wavelengths but by reflecting other colors of shorter wavelengths (Chen et al., 2020; Chittka & Waser, 1997). White flowers are also likely to be perceived differently by humans and flower‐visiting insects (Bischoff et al., 2013; Kevan et al., 1996). Despite the setup and pooled analysis of the present study being far from optimal for detecting flower color preferences, there seemed to be systematic bias from flower color that affect pan trap catches.

### 4.3 Colors of pan traps

In the present study, we did not separate catches according to the color of the pan traps, mainly because that is how such traps are currently used, but also because numbers caught in many cases would be too low to allow analyses. There is, nevertheless, evidence that different species and taxonomical groups show color preferences (Abrahamczyk et al., 2010; Campbell & Hanula, 2007; Moreira et al., 2016; Toler et al., 2005; Wilhelmsson, 2014; Wilson et al., 2008). For example, compared with other color yellow pan traps catch more Syrphidae (Wilhelmsson, 2014) and Vespoidea (Abrahamczyk et al., 2010, Moreira et al., 2016; but cf Figure 2 above, where blue caught more than yellow). On the other hand, blue pan traps have been reported to attract bees (Campbell & Hanula, 2007; Moreira et al., 2016; Toler et al., 2005). In the present study, solitary Apoidea and Syrphidae seemed to prefer yellow traps while Lepturinae avoided them (Figure 2). However, even if the total catches of these groups differed among differently colored pans, preference is doubtful to infer as catches may be influenced by amount and color of surrounding flowers (see above). So, a cautious conclusion is that pan trap studies cannot contribute to knowledge about color preferences unless it can be established that catches are unaffected by flowers in the vicinity of traps. Instead, we believe more elaborate designs are needed to further our understating of flower color preferences (e.g., Campbell et al., 2010; Reverté et al., 2016).

Flower color preferences are likely to further complicate the assumption that pan trap catches increase with insect abundance. In addition to flower color preferences, there might also be preferences for individual plant species (e.g., Hall, 2016; Mossberg & Cederberg, 2012; Pettersson & Sjödin, 2000; Söderström, 2013), social learning (e.g., local enhancement, Leadbeater & Chittka, 2007), or communication within social species (von Frisch & Chadwick, 1967) affecting catches. To further complicate the relationship between insect density, pan trap catches and flower density and color, insects can learn to visit other colors of the flowers than the innate color preference if it is rewarding (Gumbert, 2000).

### 4.4 Spatial scales

Ecological processes work on different temporal and spatial scales, and both vary among insect species (e.g., Bergman et al., 2012, 2018). A relevant process on clear‐cuts, that is, short‐term openings in forests, is dispersal (Ibbe et al., 2011; Viljur & Teder, 2018). If taxa move relatively freely over larger areas, they would be distributed according to floral resources, with lower densities in areas with few or little food to offer. Hence, dispersal would cause differentiation by floral abundance among as well as within clear‐cuts. If species do not move much, on the other hand, there is less opportunity for an insect/flower relationship to manifest itself among clear‐cuts, but there might still be an accumulation of individuals in flower‐rich spots within a clear‐cut. So, we assumed that if a negative bias exists, it would mainly be visible in data from individual trap groups rather than on the clear‐cut scale. Furthermore, the statistical power of data from the trap scale (48 trap groups) was larger than on the clear‐cut scale (12 clear‐cuts), also suggesting that a bias would be most pronounced in data from the trap scale. However, our data did not support this assumption. Rather the clear‐cut scale had larger negative bias than the trap‐scale data. Social Apoidea has the greatest dispersal ability but few consistent patterns. At the trap scale, there was often a tendency for a positive bias (see above). During the August sampling, when their population densities must have peaked, there was a strong negative bias for the clear‐cut scale. So, on balance, we conclude that a negative bias was apparent on both spatial scales considered.

### 4.5 Aggregate species‐wise data?

Another issue of general interest is whether to aim for species‐wise data or aggregated data when monitoring the pollinator fauna. With the numerous insect species involved, we should assume differing degrees of bias among them. Aggregating such data is likely to make it less likely to detect underlying insect population changes. For example, combining the six taxa considered in the current study would not have been very useful because of different population densities, flower color preferences, flight periods, and degree of bias. So, genus‐ or species‐wise analyses seem more promising but, due to statistical power, also requires collecting more specimens.

## 5 CONCLUSION

Overall, our study documented a dominance of negative bias, that is, less catches in pan traps with more flowers surrounding them. This pattern differed among taxa and often varied depending on timing of sampling, flower colors and spatial scale considered. The prevalent pattern of decreasing catches with increasing flowers presents a challenge when interpreting pan trap catches. A simple system to adjust for bias seems, given its variability, unlikely to work. Given the prevalence of negative bias in pan trap catches, the method seems less suited to evaluate differences when gradients in flower density are large (as when comparing sites or the effect of conservation intervention). Based on the same reasoning, pan‐trapping is expected to be less subject to negative bias when gradients are short, for example, when monitoring a population or assemblage.

## CONFLICT OF INTEREST

There are no conflicts of interest in this study.

## AUTHOR CONTRIBUTIONS

**Lars Westerberg:** Conceptualization (equal); data curation (lead); formal analysis (lead); methodology (equal); writing – original draft (supporting); writing – review and editing (equal). **Hilda‐Linn Berglund:** Conceptualization (equal); data curation (supporting); investigation (lead); methodology (equal); writing – original draft (lead); writing – review and editing (equal). **Dennis Jonason:** Conceptualization (equal); investigation (supporting); methodology (equal); project administration (equal); writing – original draft (supporting); writing – review and editing (equal). **Per Milberg:** Conceptualization (equal); data curation (equal); formal analysis (equal); methodology (equal); writing – original draft (lead); writing – review and editing (equal).

## RESEARCH INVOLVING ANIMALS

This study involves trapping insects for which no permit is needed in Sweden.

## Data Availability

https://doi.org/10.5061/dryad.wm37pvmmd

## ENDNOTE

^1^ Unpublished data using netting in transects in August in the current study system (cf Berglund & Milberg 2019) established a positive relationship between catches on clear‐cuts and their flower frequency.
